# SOFA Score as a Reliable Tool to Detect High Risk for Venous Thrombosis in Patients With Critical Stage SARS-CoV-2

**DOI:** 10.3389/fcvm.2021.729298

**Published:** 2021-10-28

**Authors:** Giorgio Prouse, Ludovica Ettorre, Francesco Mongelli, Daniela Demundo, Jos C. van den Berg, Carola Catanese, Luca Fumagalli, Corrado Usai, Luca Spinedi, Francesca Riva, Maria Vittoria Bertoni, Luca Giovannacci

**Affiliations:** ^1^Service of Vascular Surgery, Centro Vascolare Ticino, Ospedali Regionali di Lugano, Bellinzona e Locarno, Switzerland; ^2^Department of Surgery, Ospedale Regionale di Lugano, Lugano, Switzerland; ^3^Istituto di Imaging Della Svizzera Italiana, Ente Ospedaliero Cantonale, Lugano, Switzerland; ^4^Service of Angiology, Ospedale Regionale di Locarno, Locarno, Switzerland

**Keywords:** SARS-CoV-2, venous thromboembolism, deep vein thrombosis (DVT), anticoagulation (AC), SOFA score, SIC score, D-dimer (DD)

## Abstract

**Background:** Severe acute respiratory syndrome from coronavirus-2 (SARS-CoV-2) has been associated with an increased risk of venous thromboembolism (VTE). Different anticoagulation protocols have been applied in several studies in the absence of clear evidence. A reliable deep venous thrombosis (DVT) indicator in critical patients with SARS-CoV-2 could guide the anticoagulation treatment; however, it has not yet been identified, and clinical applicability of the most common markers is debatable. The aim of our study was to determine the actual incidence of DVT in critically ill SARS-CoV-2 patients and to find a reliable tool to identify patients who might benefit from therapeutic-intensity anticoagulation.

**Methods:** From March 1, 2020 to May 31, 2020, all patients admitted to the intensive care unit (ICU) for SARS-CoV-2 at Ospedale Regionale di Locarno, Locarno, Switzerland, were prospectively enrolled and screened daily with ultrasound for DVT. Following international consensus, a higher-intensity thromboprophylaxis was administered to all patients who were not at increased risk for bleeding. Sepsis-induced coagulopathy (SIC) and sequential organ failure assessment (SOFA) scores were calculated and time-to-DVT event in a COX proportional-hazard regression model was performed. A receiver operating characteristic (ROC) curve was used to determine sensitivity and specificity and the Youden's Index to establish the best threshold.

**Results:** A total of 96 patients were enrolled. Deep venous thrombosis was detected in 37% of patients. Sepsis-induced coagulopathy and SOFA scores were both correlated to DVT. A SIC score of 1 vs. ≥2 showed a close association with DVT, with sensitivity, specificity, and positive and negative predictive values of 90.0, 48.1, and 49.1, and 89.7%, respectively. Most significantly though, a SOFA score of 1 or 2 points was shown to be the most accurate value in predicting the absence of DVT, indicating no need for therapeutic-intensity anticoagulation. Its sensitivity, specificity, and positive and negative predictive values were 87.9, 100, and 100, and 93.7%, respectively. The D-dimer test showed lower sensitivity and specificity whereas platelet count and aPTT were not found to be correlated to DVT.

**Conclusions:** Patients with SOFA scores of 1 or 2 are at low risk of developing DVT and do not require therapeutic-intensity anticoagulation. Conversely, patients with scores ≥3 are at high risk of developing DVT.

## Introduction

The link between a severe inflammatory state and coagulopathy has been established (1). This is particularly true for severe acute respiratory syndrome from coronavirus-2 (SARS-CoV-2), in which the severe acute respiratory syndrome has been consistently linked to an increased risk of venous thromboembolism (VTE) with endothelial dysfunction potentially playing a significant additional role (2–4). Venous thromboembolism has been associated with unfavorable outcomes, with some reports describing up to 40% mortality (5). This is why several different anticoagulation protocols have been suggested in a widespread effort among scientists worldwide (6, 7). Most of these protocols, however, lack validation and are based on studies that adopted inconsistent prophylactic regimens, especially throughout the early phases of the pandemic (5, 8, 9). Despite widespread use of regular and higher-intensity thromboprophylaxis in severe cases in the later phases of the pandemic, a high incidence of thrombotic events was still observed (4, 10–12). This suggests that there may be a subgroup of critical SARS-Co-V2 patients who might benefit from therapeutic-intensity anticoagulation before the onset of thrombotic complications. To this end, although many hematological manifestations have been described in patients affected by SARS-CoV-2 (13), there still is no consensus on which are most effective to predict deep vein thrombosis (DVT) and pulmonary embolism (PE).

Our aim is to determine the parameters that can be best used to detect critical patients at high-risk for venous thrombosis and PE.

## Methods

### Study Design and Enrollment

This study was approved by the ethics committee (Comitato Etico Cantonale del Ticino, Switzerland, BASEC 2020-01354 CE 3659). All patients requiring admission to the intensive care unit (ICU) due to Covid-19 infection at Ospedale Regionale di Locarno, Locarno, Switzerland, between March 1, 2020, and May 31, 2020, were prospectively included. No patients were excluded. During the Covid-19 outbreak, this hospital has been identified as the designated hospital, treating all Covid-19 patients referred to the public hospitals network in southern Switzerland. Covid-19 infection was diagnosed with either the Xpert®X Press SARS-CoV-2 or the Viasure SARS-CoV-2 S gene. In-house PCR testing was conducted on all non-nasopharyngeal specimens with Roche reagents and primers (TIB Molbiol) using Applied Biosystems® 7500 Fast (ThermoFisher Scientifics). All patients underwent daily ultrasound screening of upper and lower limbs and of jugular veins bilaterally until thrombosis was identified. Only occlusive or sub occlusive thrombosis with clear mural involvement where considered. In case of prolonged need for prone position care, the jugular vein screening was not carried out. The ultrasound screening was continued after ICU discharge only in patients who underwent tracheostomy and were subsequently transferred to an intermediate care ward on mechanical ventilation.

Epidemiological, demographic, clinical, treatment, and outcome data were collected in a dataset. The primary endpoint was the incidence of DVT and the secondary endpoint was to evaluate the diagnostic power to predict DVT of platelet count, aPTT, D-dimer, INR, sepsis-induced coagulopathy (SIC) score, sequential organ failure assessment (SOFA) score, and simplified acute physiology score (SAPS II). The SOFA scores were recorded for all patients daily. The Glasgow Coma Scale evaluation was based on preintubation observation in all mechanically ventilated patients (14, 15). The SIC scores were retrospectively calculated. In patients who developed DVT, the last SIC and SOFA scores before the event were considered, whereas in patients in whom no DVT was detected, the highest scores during ICU stay were used. SAPS II score at ICU admission was used for all patients. SIC and SOFA scores were analyzed in order to find the most useful threshold to detect patients who were at high risk for DVT, and who could benefit from full-dose anticoagulation, and those who were at very low risk and who may not require full-dose treatment.

### Treatment Protocol

All patients admitted to the ICU without clinical and radiological evidence of VTE who were not on previous anticoagulation therapy and who were not considered at increased risk for hemorrhage were treated with higher-intensity thromboprophylaxis using low-molecular-weight heparin or unfractionated heparin (UFH) adapted to weight and glomerular filtration rate, as shown in Table 1. Therapeutic-intensity anticoagulation was initiated when evidence of VTE was found (Table 2).

**Table 1:** Weight and GFR adapted higher-intensity thromboprophylaxis.

**eGFR**	**Weight <80 kg**	**Weight ≥80 kg**
≥30 ml/min/1.73 m^2^	Enoxaparin 40 mg sc **2/day**	Enoxaparin 60 mg sc **2/day**
<30 ml/min/1.73 m^2^ and/or hemofiltration	Unfractioned heparin sc 5,000 UI **3/day**	

**Table 2:** Weight and GFR adapted therapeutic-intensity anticoagulation.

**eGFR**	**Weight <80 kg**	**Weight ≥80 kg**
≥30 ml/min/1.73 m^2^	Enoxaparin 60 mg sc **2/day**	Enoxaparin 80 mg sc **2/day**
<30 ml/min/1.73 m^2^	**Preferentially IV UFH**, *anti-Xa target 0.3–0.5 U/mL*	
and/or hemofiltration	Alternatively: UFH sc 15,000 UI **2/day**, *anti-Xa target 0.3–0.5 U/mL*	

Patients on previous oral anticoagulants were switched to therapeutic-intensity anticoagulation with low-molecular-weight heparin or UFH, and those at increased risk for bleeding received standard-dose thromboprophylaxis (enoxaparin, 40 mg/day or UFH, 5,000 UI/twice daily). Only patients with an absolute contraindication, such as relevant active bleeding, were excluded from antithrombotic treatment.

### Statistical Analysis

We used MedCalc Statistical Software version 19.4.0 (MedCalc Software Ltd., Ostend, Belgium; https://www.medcalc.org; 2020). Descriptive statistics were presented as absolute frequencies for categorical variables and mean with SD for continuous variables. The comparisons of dichotomous values were performed using the chi-squared test, whereas continuous variables between groups were compared using the Mann–Whitney test (16). For D-dimers, international normalized ratio, platelet count, SOFA and SAPS II scores, a receiver operating characteristic (ROC) curve (17) was used to calculate area under the ROC curve (AUC) sensitivity and specificity. The Youden's index was used to establish the best threshold on the ROC curve (18). A Cox proportional-hazards model was used to identify factors associated with time-to-DVT events, to test SIC and SOFA scores in regards to DVT and to provide hazard ratio (HR) and 95% confidence interval (CI). K-Fold Cross Validation method (K = 5) was used to create multiple validation subsets of our data sample and to assess our prediction model reliability. Subgroup analyses were performed to test the diagnostic power of SIC and SOFA scores in patients not on anticoagulation treatment prior to admission. The threshold of statistical significance was *P* < 0.05.

## Results

Of 450 patients admitted for Covid-19 infection, 96 required intensive care and were included in this study. Median age was 69.1 years (IQR 61.1–75.0); 69 (71.9%) were male; and 74 (77.1%) had at least one comorbidity. Median BMI was 29.6 kg/m^2^ (IQR 26.6–32.4), with 31.7 in the DVT group and 28.9 in the non-DVT group (*P* = 0.009). Median length of ICU stay was 19 days (IQR 12–25) in the DVT group and 9 days (IQR 3–19) in the non-DVT group (*P* = 0.004). A total of 84 patients (87.5%) required invasive mechanical ventilation. Additional details are shown in Table 3. The overall median time of DVT development after ICU admission was 12.5 days (IQR 8.5–20.0).

**Table 3:** Patient characteristics and primary clinical outcomes.

	**DVT group**	**No DVT group**	***P***
	***n*** **= 36**	***n*** **= 60**	
Age, years (IQR)	70.3 (62.5–74.6)	68.8 (60.6–75.2)	0.748
Sex, male (%)	23 (63.9)	46 (76.7)	0.179
Comorbidities			
Cardiovascular disease, *n* (%)	9 (25.0)	21 (35.0)	0.309
Hypertension, *n* (%)	14 (38.9)	35 (58.3)	0.066
Pulmonary disease, *n* (%)	5 (13.9)	9 (15.0)	0.882
Renal disease, *n* (%)	2 (5.6)	8 (13.3)	0.229
Presence of a solid tumor, *n* (%)	1 (2.8)	1 (1.7)	0.714
Diabetes, *n* (%)	7 (19.4)	17 (28.3)	0.333
Dementia, *n* (%)	1 (2.8)	1 (1.7)	0.714
Immunosuppressive status, *n* (%)	2 (5.6)	1 (1.7)	0.292
BMI, kg/m^2^ (IQR)	31.7 (29.7–39.1)	28.9 (25.8–30.4)	**0.009**
Active smoking, *n* (%)	3/18 (16.7)	1/16 (6.2)	0.354
Vital signs on admission			
Systolic blood pressure, mmHg (IQR)	136 (123–148)	129 (120–141)	0.210
Hearth rate, BPM (IQR)	87 (74–95)	84 (71–96)	0.576
Temperature, °C (IQR)	37.6 (36.9–38.3)	37.6 (36.8–38.1)	0.759
Respiratory rate, breaths per minute (IQR)	22 (19-24)	24 (20-30)	0.161
Days positive test to ICU admission	2.0 (1.0–5.5)	3.5 (0–6.0)	0.848
Length of ICU stay, days (IQR)	19 (12–25)	9 (3–19)	**0.004**
Early Warning Score, points (IQR)	4 (3–6)	5 (3–9)	0.122
SAPS II, median (IQR)	42 (37–48)	41 (36–54)	0.762
SIC score, points (IQR)	2 (2)	2 (1–2)	**0.015**
SOFA score, points (IQR)	5 (3–7)	1 (1)	**<0.001**
INR, median (IQR)	1.2 (1.1–1.25)	1.2 (1.1–1.3)	0.638
LDH_max_, median (IQR)	790.5 (604–1115)	722 (486–870)	0.112
ALT_max_, median (IQR)	50 (30–103)	42 (34–78)	0.501
AST_max_, median (IQR)	76 (55–121)	44 (31–71)	**0.010**
aPTT, sec. (IQR)	33.5 (30–46.25)	35 (30–55.5)	0.356
PLT, n x 10^9^/L (IQR)	328 (236–552)	403.5 (217–559)	0.510
Mechanical ventilation, n (%)	34 (94.4)	50 (83.3)	0.113
Anticoagulation regimens			
- None, *n* (%)	0	1 (1.7)	**0.032**
- Simple prophylaxis, *n* (%)	28 (77.8)	29 (48.3)	
- High prophylaxis, *n* (%)	4 (11.1)	20 (33.3)	
- Anticoagulation, *n* (%)	4 (11.1)	10 (16.7)	
Mortality, *n* (%)	12 (33.3%)	24 (38.3%)	0.354

*Continue variables are expressed as median with interquartile range (IQR) in parentheses, frequencies are expressed as absolute number with percentage in parentheses. DVT, deep vein thrombosis; BPM, beats per minute; °C, Celsius degrees; BMI, body mass index; SAPS, simplified acute physiology score; SIC, sepsis induced coagulopathy; SOFA, sequential organ failure assessment; INR, International Normalized Ratio; aPTT, activated partial thromboplastin time; PLT, platelet count; ICU, intensive care unit. Bold values are the statistically significative ones*.

**Figure 1:**
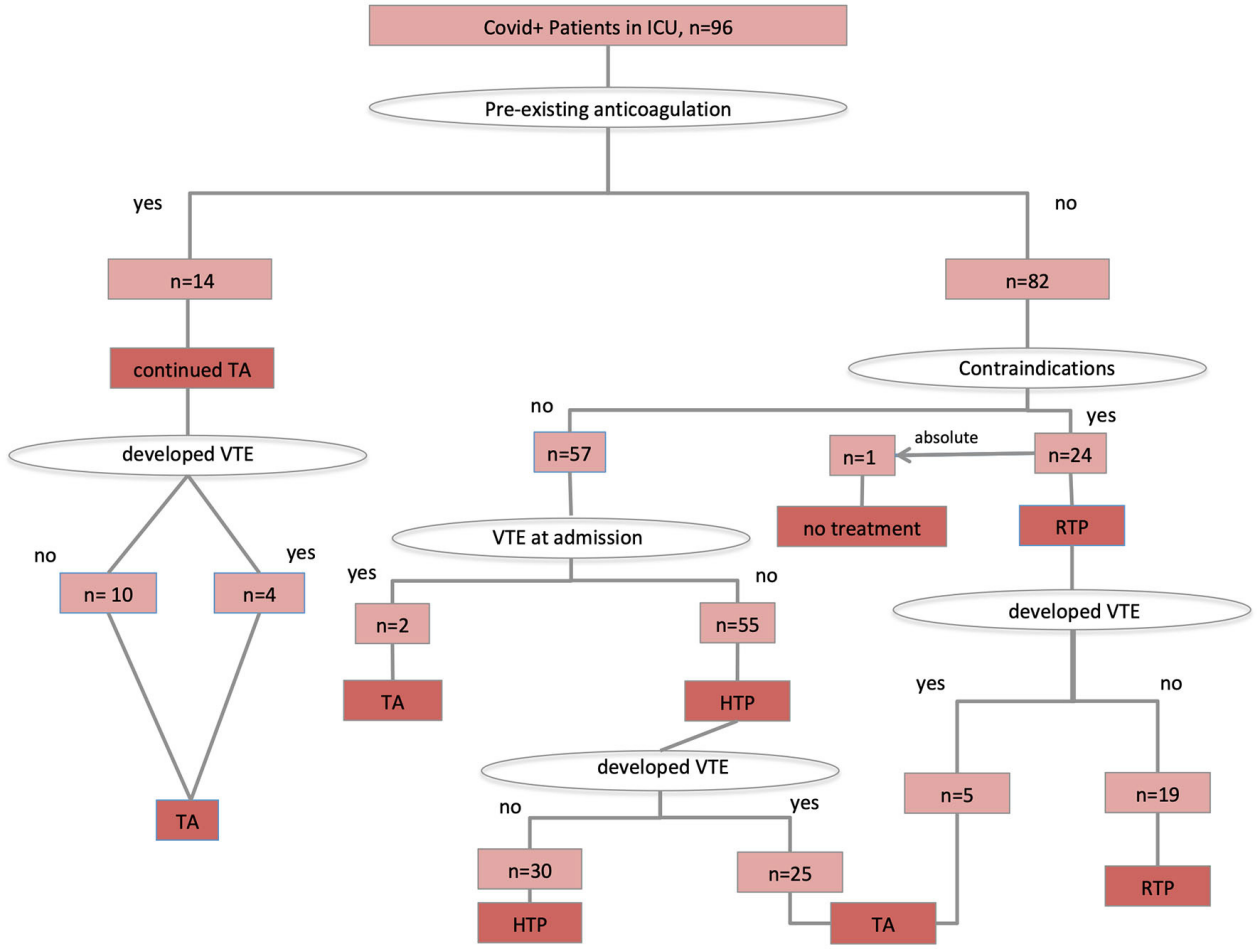
Antithrombotic treatment protocol application. TA, therapeutic anticoagulation; HTP, higher-intensity thromboprophylaxis; RTP, regular-intensity thromboprophylaxis.

Ultrasound screening carried out in all critical SARS CoV-2 patients detected DVT in 37% of cases. A total of 55 patients were on higher-intensity thromboprophylaxis, whereas 16 patients were on therapeutic-intensity anticoagulation, and 24 patients on regular-dose thromboprophylaxis because of increased risk for bleeding. One patient presented with concomitant subdural hematoma at admission and received no antithrombotic treatment. Details on anticoagulation treatment and prophylaxis are shown in the Figure 1.

Nine patients had bleeding events, three were major bleedings of which two were fatal. Eight of these nine patients were on therapeutic-intensity anticoagulation and one was on higher-intensity prophylaxis.

**Figure 2:**
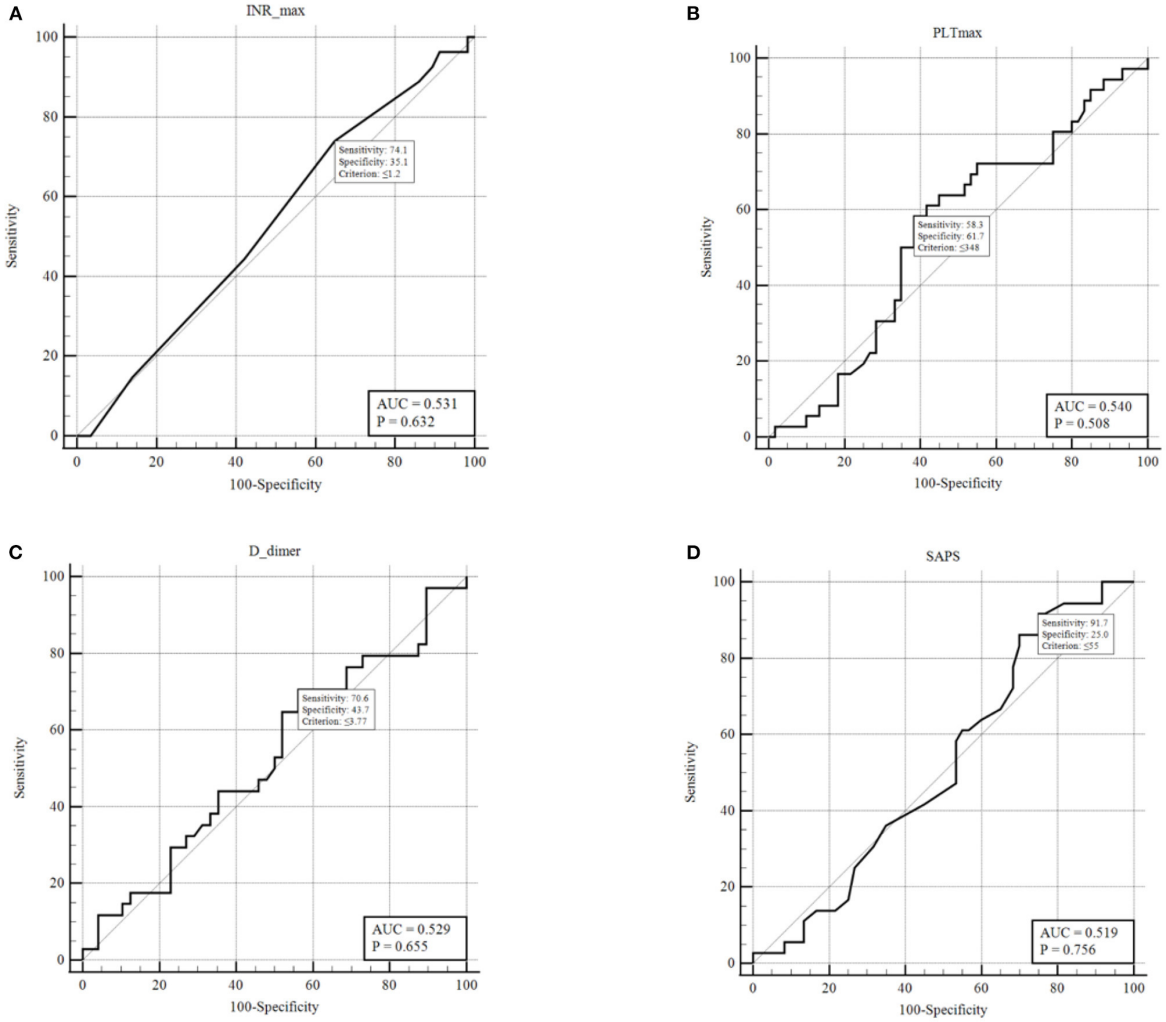
ROC curves for: **(A)** INR last value pre-DVT or highest value if no DVT; **(B)** PLT last value pre-DVT or highest value if no DVT; **(C)** D-dimers last value pre-DVT or highest value if no DVT; **(D)** SAPS II score within 24 h from ICU admission.

**Figure 3:**
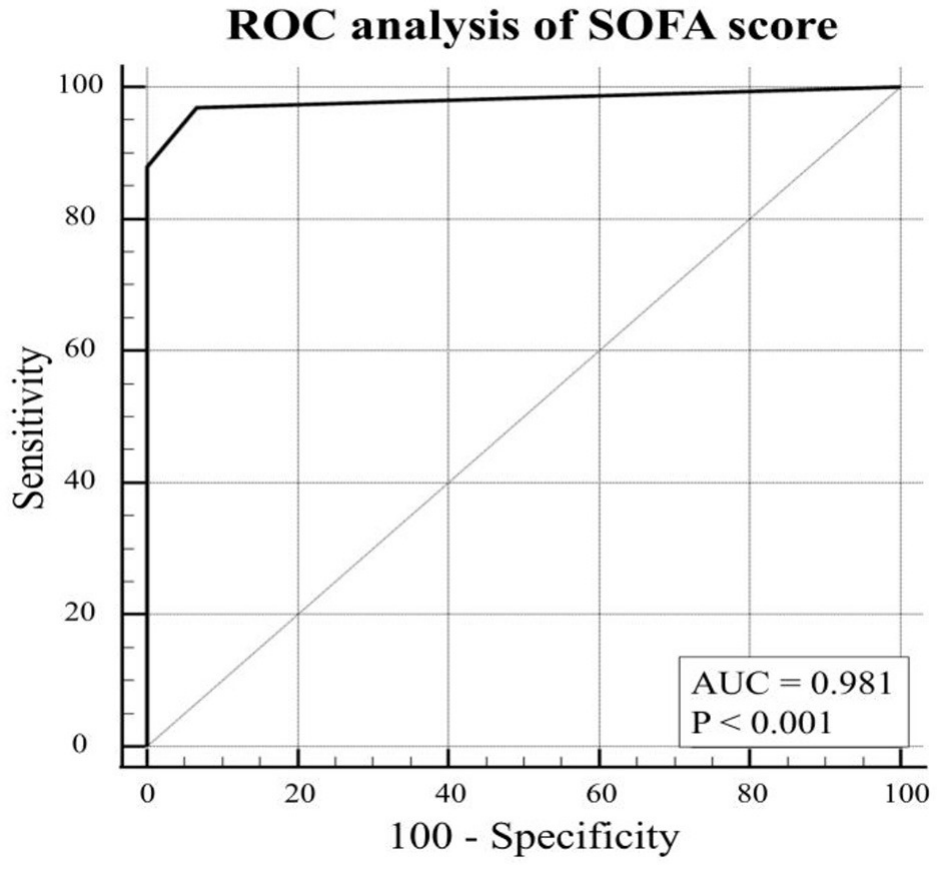
ROC curve for SOFA score last value pre-DVT or highest value if no DVT.

The ROC analysis resulted not significant in predicting the presence of DVT for D-dimers, platelet count, International normalized ratio, and SAPS II score (Figures 2A–D). Conversely, SOFA score showed an AUC of 0.981 (*p* < 0.001) (Figure 3). With a SOFA score threshold ≥2 points, we found the test to have a sensitivity of 97.0% and a specificity of 93.3%, while with a threshold ≥3 points the sensitivity was 87.9% and specificity 100%. SIC scores ≥2 showed the best association with DVT, with sensitivity, specificity, and positive and negative predictive values of 90.0, 48.1, and 49.1 and 89.7%, respectively. There were no patients with a SIC score of 0. The K-Fold Cross Validation Method confirmed the high diagnostic power of the SOFA score prediction model. Sensitivity, specificity and positive and negative predictive values were, respectively, 88.3, 100.0, 93.8, and 100.0%.

**Figure 4:**
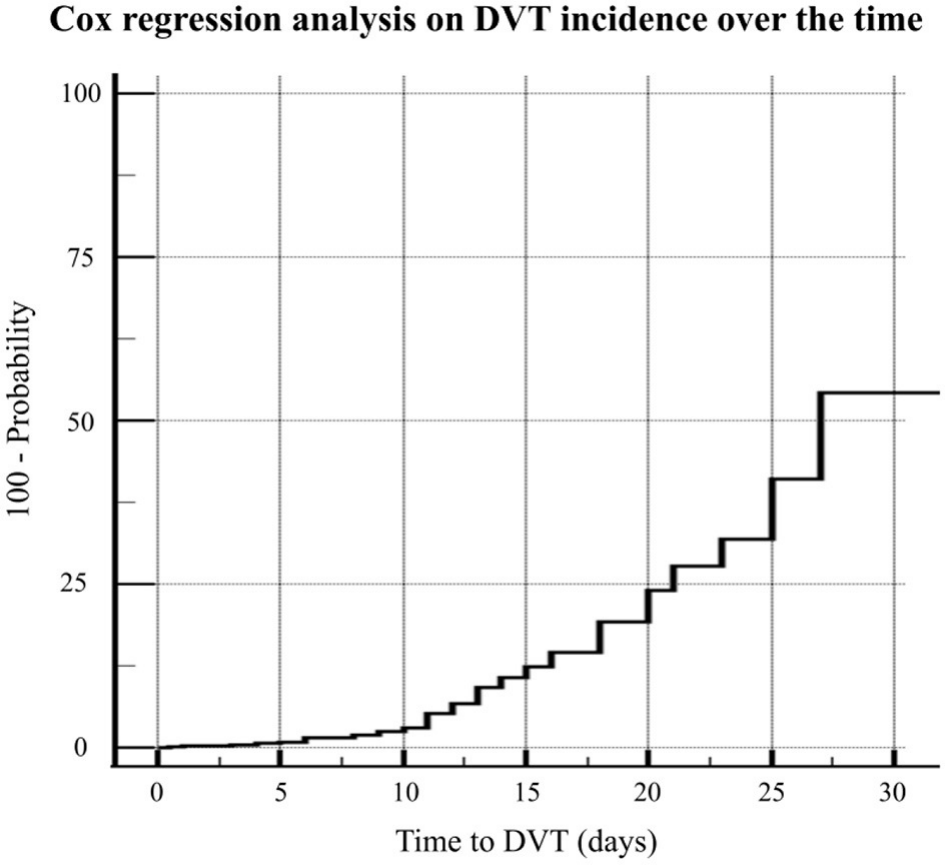
The Cox proportional-hazard regression analysis including eight different factors potentially associated to time-to-DVT event (i.e., age, sex, BMI, SOFA score, comorbidities, anticoagulation intensity, d-dimer level, length of ICU stay).

The Cox proportional-hazard regression analysis was carried out including eight different factors potentially associated to time-to-DVT event (i.e., age, sex, BMI, SOFA score, comorbidities, anticoagulation intensity, d-dimer level, length of ICU stay). In the regression model four factors associated with DVT were retained: age (HR 0.954, 95%CI 0.914–0.997, *p* = 0.034), sex (HR 0.400, 95%CI 0.164–0.976, *p* = 0.044), SOFA score (HR 1.871, 95%CI 1.574–2.225, *p* < 0.001), and anticoagulation intensity (HR 0.407, 95%CI 0.219–0.758, *p* = 0.005) (Figure 4).

## Discussion

Although the danger associated with hypercoagulability in patients with severe SARS-Co-V2 has been observed repeatedly and is well-accepted, three fundamental questions remain uncertain: What is the real incidence of DVT and PE in this subset of patients?; Which prophylactic and anticoagulation strategies should be applied?; and Which are the most reliable markers that allow detection of patients who may benefit from anticoagulation treatment while avoiding overtreatment in patients at very low risk of developing VTE?. Numbers reported in different studies to address the first question are of limited value to determine the actual incidence of DVT or PE, because most diagnostic tests were carried out only in patients who showed clinical symptoms (2). This approach is understandable given the fact that extensive screening of COVID patients for DVT potentially exposes operators to an increased risk of infection if serious precautions are not taken. During this medical emergency, when resources are often depleted, it could be argued that carrying out routine screening of all critical patients poses too high a risk of infecting medical staff and is too time consuming (19). Moreover, in terms of cost effectiveness, an analysis of systematic daily ultrasound screening should be carried out (20). Conversely, the difficulty in detecting minor PE in the subset of severe SARS-CoV-2 patients subjected to mechanical ventilation potentially leaves some events unrecognized. It is therefore impossible to determine the real incidence of PE or *in situ* pulmonary artery thrombosis. In some case series, despite VTE was not clinically suspected before death, an occlusion of the pulmonary artery has been found in post mortem examination (10, 21). We decided to prospectively gather data from daily ultrasound screenings using a dedicated team of radiologists and angiologists. Performing a daily duplex ultrasound on all the patients is an advantage of our study. This allowed us to detect almost all patients with DVT, revealing the actual incidence of this complication. After discharge from the ICU the daily screening was continued only in those patients who were transferred to intermediate care for mechanical ventilation through tracheostomy. Although it is possible that some patients developed DVT at a later time, our study focused only on those events triggered by the critical stage during organ function support.

The team that carried out the examinations took all necessary precautions. One month after the last patient was discharged, all members of the team where tested serologically. One staff member of six tested positive, and none had presented any symptoms. Venous thrombosis was detected in a very high number (37%) of these patients with several among them remaining asymptomatic for VTE. This is consistent with reports of the high prevalence of thrombosis at all levels, including central lines, dialysis catheters, and extracorporeal membrane oxygenation (2, 5).

The second question regarding the anticoagulation treatment has been addressed by many study groups and is still widely debated. Most studies that reported data from the early stages of the pandemic included series of patients who had undergone different prophylactic and anticoagulation strategies, making results poorly comparable (5, 8, 22). Early in the pandemic one relevant study showed a benefit of anticoagulation treatment in terms of 28-day survival in patients with a six-fold D-dimer elevation, regardless of existing VTE. The results though were biased by a high percentage of patients who were not treated with thromboprophylaxis (8). A review of randomized trials comparing full anticoagulation to standard-dose and higher-intensity thromboprophylaxis treatments in Covid-19 patients identified 20 ongoing trials. The review showed trials to be of low quality and heterogeneous (23) with a mix of different outcomes and lack of differentiation regarding disease severity. A subsequent systematic review found slight evidence that therapeutic anticoagulation may improve survival amongst mechanically ventilated Covid-19 patients (24). More recently, two large randomized controlled trials compared prophylaxis to therapeutic anticoagulation in non-critical (25) and in critical (26) Sars-Cov-2 patients. The first one found an advantage in terms of survival amongst non-critical patients treated with therapeutic anticoagulation whereas the latter did not show an increase in survival and in number of days free of cardiovascular or respiratory organ support with therapeutic anticoagulation. Additionally, some recent studies suggest a platelet hyperactivation as contributing to the pro-thrombotic state occurring in Covid-19 infection (27, 28).

In our center, an aggressive treatment strategy with higher-intensity prophylaxis, when feasible and therapeutic anticoagulation as soon as DVT was detected was applied throughout. This approach has been recommended by several study groups (6), although it is currently still not validated by clear evidence (23).

Despite this more aggressive approach, there was a 37% prevalence of DVT, indicating that a subgroup of patients might benefit from therapeutic anticoagulation. This finding is confirmed by several recently published series that included patients admitted to the ICU (3, 29, 30).

The third question is generated by the necessity to detect those critical patients who may benefit from therapeutic anticoagulation, ideally before the onset of life-threatening VTE events, while avoiding overtreatment in all other critical patients. In fact, some studies have shown an increased risk of major bleedings in COVID-19 patients treated with therapeutic anticoagulation (31). Furthermore, the randomized controlled trial on critical patients by the REMAP-CAP, ACTIV-4a study group (26) was interrupted for futility of therapeutic anticoagulation over regular thromboprophylaxis, confirming that therapeutic anticoagulation should not be routinely administered to all critical patients. The contradicting results of other previous studies (24) suggest there may be a subgroup of critical patients that still might benefit from therapeutic anticoagulation.

Although several hematological alterations have been described in critical SARS-CoV-2 patients, only a few of them have been suggested to be useful predictors for survival and a reliable indicator with an accepted threshold for increased risk of VTE events in patients with severe SARS-CoV-2 has not yet been established.

In this study, we analyzed D-dimers, platelet count, aPTT, SIC, and SOFA scores. The SOFA score is a valid predictor of in-hospital mortality (32), identifying high-risk patients using basic clinical criteria (33). The SIC score also takes platelet count and INR values into account. It is a validated score to determine a high risk of disseminated intravascular coagulation among septic patients and may identify those who could benefit from anticoagulant therapy (34–36).

Regarding the D-dimer analysis, the Youden index determined a threshold of 3.77 mg/ml with associated sensitivity and specificity of 73.5 and 44.7%, respectively. Not treating patients with a D-dimer below this threshold will leave a significant number of patients untreated who are at high risk of developing DVT. Conversely, lowering this threshold will cause most critical patients to be treated with anticoagulation, given the prevalence of elevated D-dimers observed. It has been extensively shown that D-dimer levels are correlated to mortality in all hospitalized COVID-19 patients (37, 38), yet it has not proven in our study to be a useful indicator for the risk of DVT or to determine patients who should undergo full anticoagulation. Tang et al. (8) showed a benefit of anticoagulation treatment in terms of 28-day survival in patients with a six-fold D-dimer elevation (>3 mg/L), regardless of existing VTE. These results are not comparable with results reported in several other series, including ours, because of a high percentage of patients who were not treated with any thromboprophylaxis (78%).

The ROC curves for platelet count and aPTT exhibited an AUC of 0.54 and 0.57, respectively, indicating they must not be used as markers to detect high-risk or low-risk patients. The analysis of SIC scores showed a significant correlation with DVT with *P* = 0.0002. Although, when analyzing by grouping to determine a useful threshold, we found that SIC scores 1 + 2 vs. 3 + 4 had a low sensitivity (20%) and a specificity of 77.7% for DVT. A more significant grouping was found with SIC score 1 vs. ≥2, which had a high sensitivity (90%) but a specificity of only 48.1%, potentially exposing several patients to unnecessary therapeutic-intensity anticoagulation.

A significant correlation of SOFA score with DVT (*P* < 0.0001) was also found. By pooling patients with a SOFA score 1 vs. scores ≥2, we found the test to have a sensitivity of 96% and a specificity of 93%. Conversely, by grouping scores 1 + 2 vs. ≥3, the sensitivity is reduced to 87.9%, but the test displayed a specificity of 100%. This finding allows us to determine that patients with SOFA scores 1 and 2 are unlikely (93.8%) to develop DVT and may therefore be treated with thromboprophylaxis only. Conversely, patients with a SOFA score ≥3 are at high risk of developing thrombosis-related complications and may represent a subgroup that could benefit from therapeutic anticoagulation. We find this threshold to be the most useful from a clinical point of view because 69% of our patients were included in the SOFA 1 + 2 pool. This allows to withhold full anticoagulation treatment from a relevant number of critical patients, while determining a group of patients who might benefit from therapeutic anticoagulation treatment and for whom the increased risk of hemorrhage is justified. Since a relatively high number of patients (14) were already on therapeutic anticoagulation prior to admission, a subgroup analysis was performed. It showed no relevant difference to the main analysis and confirms validity of our findings in patients who were not previously on anticoagulation. The role of higher-intensity thromboprophylaxis, conversely, remains uncertain. Although a strict protocol was applied, excluding only patients at increased risk for bleeding from this treatment regimen, the prevalence of thrombosis was comparable to that described in several other studies that applied lower regimen prophylaxis. Several prospective studies are being conducted to determine the effectiveness and safety of higher-intensity prophylactic regimens.

This study has some limitations. The first one is the retrospective analysis on prospectively collected data. Furthermore, the relatively small number of patients does not allow to perform finer subgroup analyses and may limit the overall quality of evidence provided. A potential bias could be represented by the relatively large number of patients on full anticoagulation treatment prior to admission, though statistical analysis in patients without previous anticoagulation showed no relevant difference. A further source of potential bias is the operator-dependent variability of the ultrasound screening. The exams were all performed by a relatively small team (six) of trained radiologists and angiologists to limit the variability. A few exams were carried out in very difficult conditions, potentially leaving some events undetected. Finally, PE events were not included in the analysis because there is not a reliable method to detect all events of PE and of *in situ* pulmonary artery thrombosis in mechanically ventilated patients. This potentially leaves some patients without DVT but who developed PE unrecognized in our study.

## Conclusions

Both SIC score and SOFA score are significantly correlated with DVT in critical stages of SARS-CoV-2. Patients with SOFA scores 1 and 2 are at low risk of developing DVT and could avoid therapeutic-intensity anticoagulation. Conversely, patients with scores ≥3 are at high risk of DVT.

## Data Availability Statement

The raw data supporting the conclusions of this article will be made available by the authors, without undue reservation.

## Ethics Statement

The studies involving human participants were reviewed and approved by Comitato Etico Cantonale del Ticino, Switzerland, BASEC 2020-01354 CE 3659. Written informed consent for participation was not required for this study in accordance with the national legislation and the institutional requirements.

## Author Contributions

LE, MB, FR, and DD did the literature search. GP, LG, LE, FM, and DD were responsible for study design. LS, MB, FR, CU, and CC collected the data. GP, FM, LG, LE, JB, and LS accomplished data analysis and interpretation. Drafting by LE, FM, CU, FR, CC, and MB. Critical revisions under responsibility of GP, LE, FM, LG, and JB. All authors contributed in correcting and approving the final manuscript.

## Funding

The study was funded by the Scientific Fund of the Centro Vascolare Ticino, Switzerland. The funder had no role in the study design, in the collection, analysis, interpretation of data, in the writing of the report, and in the decision to submit the paper for publication. The funder enabled open access publication.

## Conflict of Interest

The authors declare that the research was conducted in the absence of any commercial or financial relationships that could be construed as a potential conflict of interest.

## Publisher's Note

All claims expressed in this article are solely those of the authors and do not necessarily represent those of their affiliated organizations, or those of the publisher, the editors and the reviewers. Any product that may be evaluated in this article, or claim that may be made by its manufacturer, is not guaranteed or endorsed by the publisher.

## Abbreviations

SARS-CoV-2: severe acute respiratory syndrome coronavirus 2
VTE: venous thromboembolism
DVT: deep vein thrombosis
PE: pulmonary embolism
aPTT: activated partial thromboplastine time
INR: international normalized ratio
PLT: platelet count
SIC: sepsis induced coagulopathy
SOFA: sequential organ failure assessment
SAPS II: simplified acute physiology score
ICU: intensive care unit
UFH: unfractionated heparin
ROC: receiver operating characteristic
AUC: area under curve
OR: odds ratio
CI: confidence interval
BMI: body mass index
IQR: interquartile range.
